# A quantitative analysis of artificial intelligence research in cervical cancer: a bibliometric approach utilizing CiteSpace and VOSviewer

**DOI:** 10.3389/fonc.2024.1431142

**Published:** 2024-09-03

**Authors:** Ziqi Zhao, Boqian Hu, Kun Xu, Yizhuo Jiang, Xisheng Xu, Yuliang Liu

**Affiliations:** ^1^ School of Basic Medicine, Zhejiang Chinese Medical University, Hangzhou, China; ^2^ Hebei Provincial Hospital of Traditional Chinese Medicine, Hebei University of Chinese Medicine, Shijiazhuang, Hebei, China

**Keywords:** cervical cancer, artificial intelligence, bibliometrics, CiteSpace, VOSviewer

## Abstract

**Background:**

Cervical cancer, a severe threat to women’s health, is experiencing a global increase in incidence, notably among younger demographics. With artificial intelligence (AI) making strides, its integration into medical research is expanding, particularly in cervical cancer studies. This bibliometric study aims to evaluate AI’s role, highlighting research trends and potential future directions in the field.

**Methods:**

This study systematically retrieved literature from the Web of Science Core Collection (WoSCC), employing VOSviewer and CiteSpace for analysis. This included examining collaborations and keyword co-occurrences, with a focus on the relationship between citing and cited journals and authors. A burst ranking analysis identified research hotspots based on citation frequency.

**Results:**

The study analyzed 927 articles from 2008 to 2024 by 5,299 authors across 81 regions. China, the U.S., and India were the top contributors, with key institutions like the Chinese Academy of Sciences and the NIH leading in publications. Schiffman, Mark, featured among the top authors, while Jemal, A, was the most cited. ‘Diagnostics’ and ‘IEEE Access’ stood out for publication volume and citation impact, respectively. Keywords such as ‘cervical cancer,’ ‘deep learning,’ ‘classification,’ and ‘machine learning’ were dominant. The most cited article was by Berner, ES; et al., published in 2008.

**Conclusions:**

AI’s application in cervical cancer research is expanding, with a growing scholarly community. The study suggests that AI, especially deep learning and machine learning, will remain a key research area, focusing on improving diagnostics and treatment. There is a need for increased international collaboration to maximize AI’s potential in advancing cervical cancer research and patient care.

## 1 Introduction

Cervical cancer is one of the most common malignant tumors among women, and in recent years, there has been a trend towards younger ages of onset, posing a direct threat to women’s health and life safety (1). According to data from the Global Cancer Epidemiology Database (GLOBOCAN), in 2020, there were approximately 604,000 new cases of cervical cancer worldwide, and about 342,000 deaths, ranking fourth in both incidence and mortality rates among all malignant tumors globally, highlighting cervical cancer as a serious global health issue (2). Despite certain advancements in diagnostic technology and treatment methods for cervical cancer, such as improving the accuracy of image analysis for diagnosis (3) and enhancing patient survival rates through adjuvant therapy (4), there are still limitations to current approaches. Therefore, early screening for cervical cancer patients and the optimization of treatment plans are crucial for enhancing patients’ quality of life and improving prognosis.

Artificial Intelligence (AI), a significant branch of computer science, aims to develop systems capable of emulating human intelligent behavior, performing tasks such as learning, reasoning, problem-solving, knowledge comprehension, language recognition, visual perception, natural language processing, and robotics (5). With breakthroughs in deep learning algorithms and rapid advancements in computer technology, AI has increasingly played a vital role in the medical field.

In recent years, AI has demonstrated remarkable effectiveness in medical diagnosis (6), treatment planning (7), and disease risk prediction (8) through its superior algorithms and learning mechanisms. AI systems can analyze medical imagery, such as X-rays, CT scans, and MRI images, to identify and monitor diseases (9–11). Moreover, they assist doctors in formulating personalized treatment plans that take into account the patient’s genetic background, lifestyle habits, and disease characteristics (12). In the field of surgery, AI has enhanced the precision and safety of operations through robotic surgical systems (13).

Especially in the field of cervical cancer research, the application and implementation of AI are attracting growing attention from scholars, and research in this area is showing a rapidly increasing trend. This presents researchers with the challenge of keeping up with the latest scientific findings, grasping the dynamics of research, and predicting future directions for the field.

Bibliometric analysis (14, 15), as a form of information visualization technology, reveals the knowledge structure and research dynamics within a specific academic field by summarizing and quantitatively analyzing global literature data. This method employs mathematical and statistical tools to analyze the bibliometric characteristics of literature, aiming to identify academic frontiers and research hotspots. It is an important research tool within the field of information science. Given its rigorous and objective analysis, bibliometric analysis has become a commonly used method for scholars across various disciplines to explore the development trends in their respective fields (16–18). However, there is a lack of systematic bibliometric discussion on the application of artificial intelligence in the field of cervical cancer. Based on this, the present study collects and analyzes literature from relevant databases to conduct a quantitative and qualitative analysis of the application of artificial intelligence in cervical cancer research, and visualizes the results. The study aims to provide researchers in this field with a comprehensive overview of research progress, reveal current research priorities, and offer valuable insights into future research directions.

## 2 Methods

### 2.1 Data collection and sources

To ensure the alignment of research directions with the database, this study selected the Science Citation Index Expanded (SCI-E) and the Social Sciences Citation Index (SSCI) from the Web of Science Core Collection (WoSCC) as the data sources. These two databases are renowned for their standardization and comprehensiveness, covering a wide range of publications across various fields, and are therefore frequently used for bibliometric analysis.

### 2.2 Search strategy and criteria

In order to maintain the currency of the data and to avoid the need for updates as the research progresses, this study organized two researchers to conduct a synchronized search for papers involving artificial intelligence (AI) in the field of cervical cancer. The search and data collection were efficiently completed on the same day. The collected data includes the title, keywords, abstract, authors, institutions, and references of the articles, all of which were obtained and saved in plain text format. The specific search strategy is as follows:

TS = (((cervix OR Cervical) NEAR/1 (cancer* OR tumor* OR tumor* OR disease OR lesion* OR carcinoma*)) OR “carcinoma of uterine cervix” OR “cervical neoplasms”) AND TS = (((automated OR intelligent) NEAR/1 (classification OR diagnosis OR segment* OR detect*)) OR “artificial intelligence” OR “deep learning” OR “convolutional neural network*” OR “machine learning” OR “CNNs” OR “artificial neural network*” OR “computer-aided” OR “Bayes* network*” OR “supervised learning” OR “unsupervised clustering” OR “computer-assisted” OR (deep NEAR/1 network*) OR “ensemble learning”)

**Figure 1:**
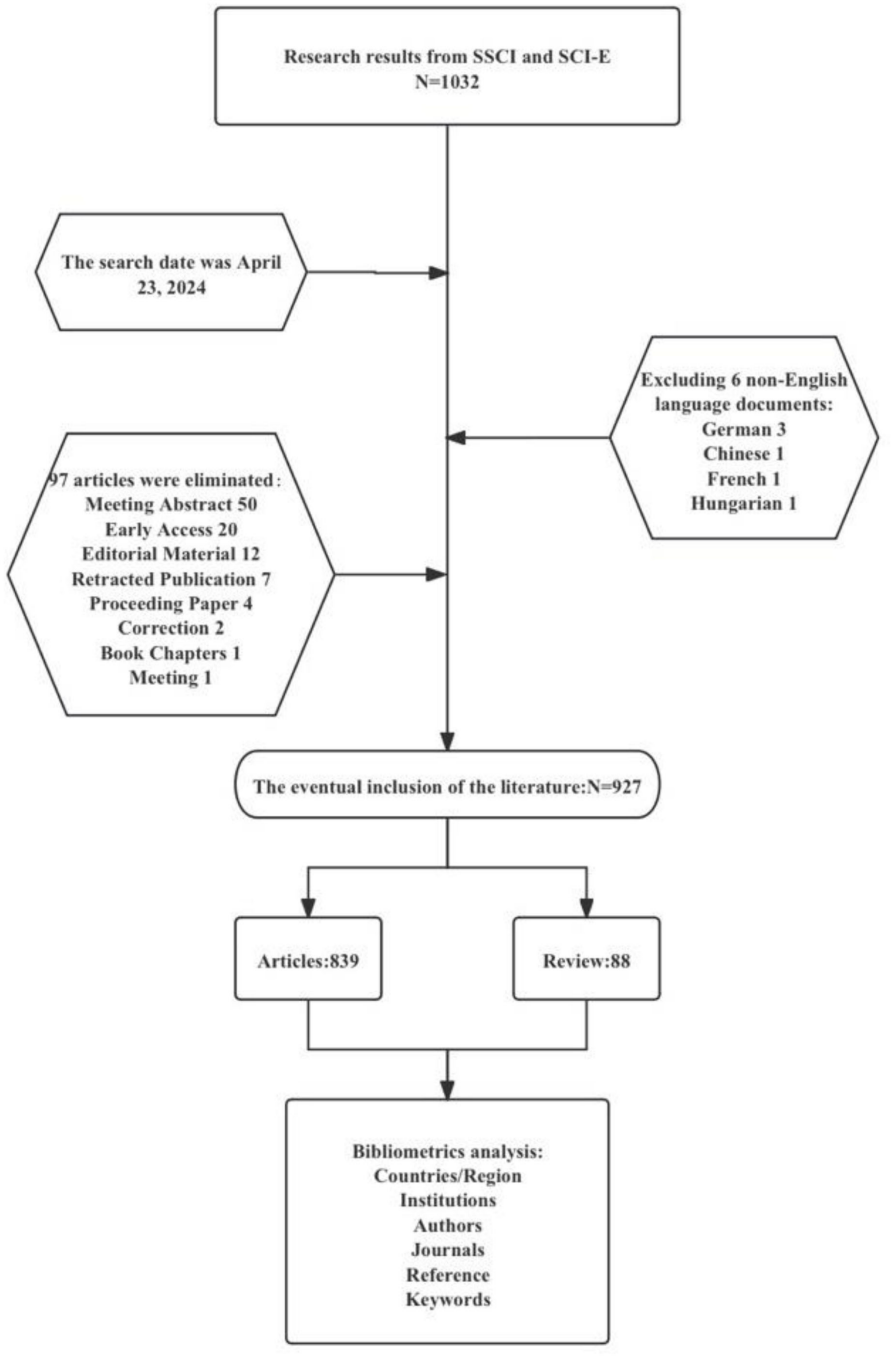
A flow chart of the retrieval process in this study.

The search was conducted on April 23, 2024, and the detailed steps of the search are displayed in **Figure 1**.

### 2.3 Manual screening process

Based on the specific requirements of this study, we designed a set of clear inclusion criteria: papers eligible for analysis must meet the following conditions: (1) the language must be English; (2) the type of article is limited to original research and review articles; (3) the research content focuses on the field of cervical cancer and involves the application of artificial intelligence technology. Through the initial search strategy, we preliminarily screened 1,027 relevant papers. Subsequently, based on the aforementioned criteria, we conducted a more meticulous manual screening. To ensure the accuracy and effectiveness of the screening results, this step was independently carried out by two researchers, who compiled and discussed any uncertain literature encountered during the screening process to determine whether it should be included in the final research scope. After this screening process, the study ultimately included 927 papers, consisting of 839 original research articles and 88 review articles.

### 2.4 Bibliometric analysis methodology

Bibliometrics, which originated in the early 20th century, is a quantitative analysis method that uses statistical techniques to systematically describe the development trends and dynamic changes in specific disciplines or research fields. In this study, we employed bibliometric tools such as VOSviewer, CiteSpace and Bibliometrix-R to conduct an in-depth visualization analysis across multiple dimensions, including countries, institutions, authors, journals, keywords, and co-citation literature.

The VOSviewer software was utilized to conduct an in-depth analysis of the collaborative relationships between countries/regions, institutions, the co-occurrence of keywords, and the collaboration among cited authors, thereby revealing the structure and dynamics of the research network. The CiteSpace software was employed to generate a dual-map overlay of journals, analyzing the relationships between cited journals and conducting a burst sorting analysis of highly cited documents and keywords to identify research hotspots. Additionally, the bibliometrix-R package was used to create a heatmap of publication volumes by country. Furthermore, we used Microsoft Excel for frequency statistics on selected analysis items, based on which tables were constructed.

## 3 Results

### 3.1 Overview of global publication trends and citations

**Figure 2:**
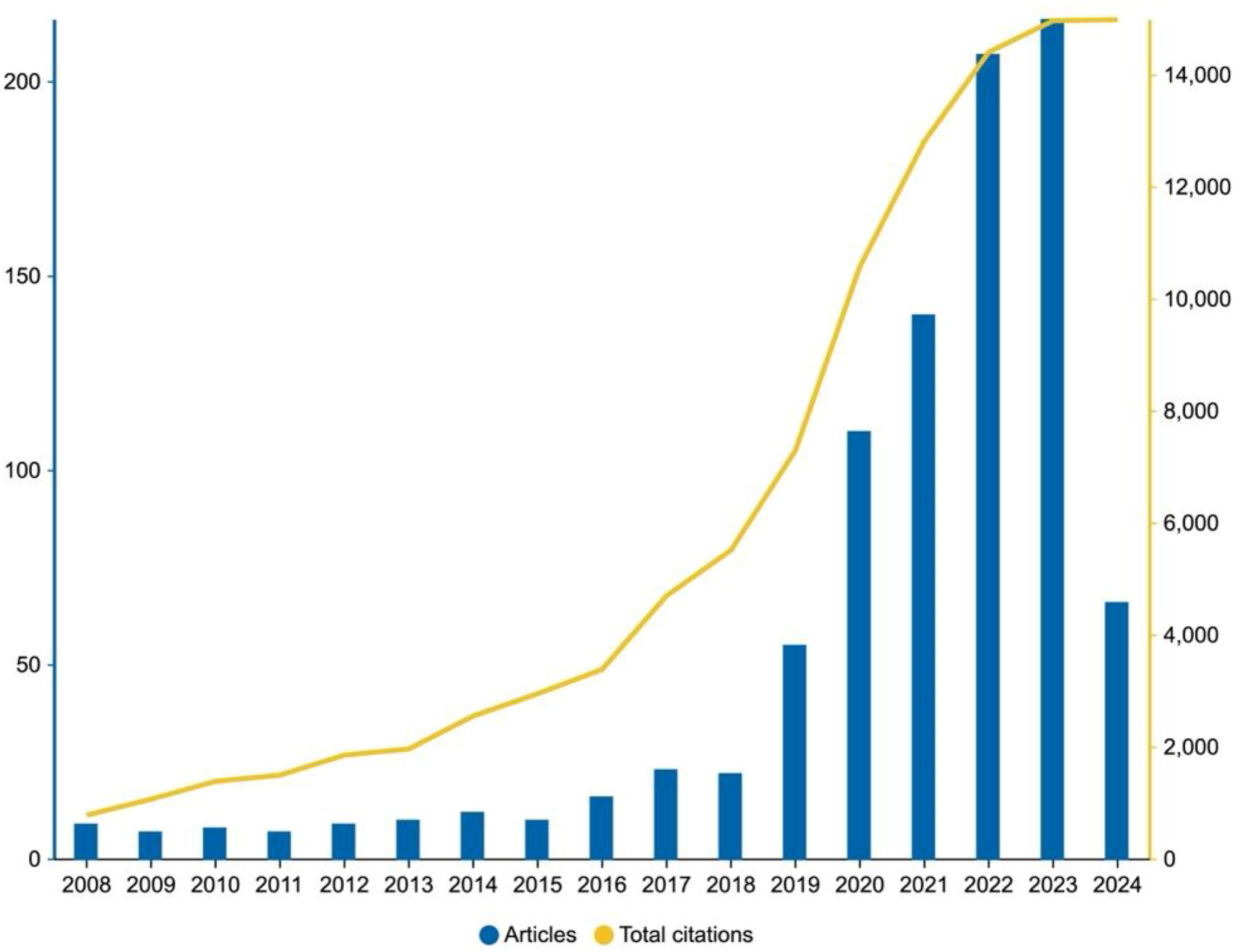
Global publications and average citation trends of artificial intelligence research on cervical cancer (2008–2024).

Through meticulous search strategies and rigorous selection processes, we have diligently curated a collection of 927 scholarly works closely associated with cervical cancer from the Web of Science Core Collection (WoSCC) database, encompassing 839 original articles and 88 comprehensive reviews (**Figure 1**). We conducted a meticulous domain statistics and summary of the included literature. Upon analysis, we found that the three fields with the highest number of publications are engineering, computer science, and oncology. Specifically, the engineering field has the highest proportion of literature, accounting for 24.488%, followed closely by the computer science field at 20.82%, and the oncology field also has a significant proportion, at 20.614%. These data not only highlight the importance of interdisciplinary collaboration in cervical cancer research with artificial intelligence but also reflect the central role of these fields in driving the development of related research. To facilitate a more intuitive understanding of the publication situation in each field for readers, we have organized the detailed statistical results in **Supplementary Table S1**. The application of artificial intelligence in cervical cancer research dates back to 2008. Although the annual publication volume was relatively low initially, it has since shown a steady growth rate of approximately 9% per year (**Figure 2**).

Especially noteworthy is the significant surge in publication volume since 2019 to the present (as of April 23, 2024), where publications from this period account for 85.7% of the total output, and the annual growth rate has skyrocketed to 76%. This dramatic increase is closely linked to the significant breakthroughs in machine learning and deep learning technologies in recent years (19, 20). Given the current trajectory, we anticipate that the number of publications in 2024 will reach new heights.

Consistent with the rise in publication volume, the total citation count has also exhibited a continuous upward trend, becoming particularly pronounced after 2018. This not only signifies the expansion of the research field’s impact but also reflects the sustained growing interest and emphasis that the academic community places on cervical cancer research.

### 3.2 Productive countries/regions analysis

**Figure 3:**
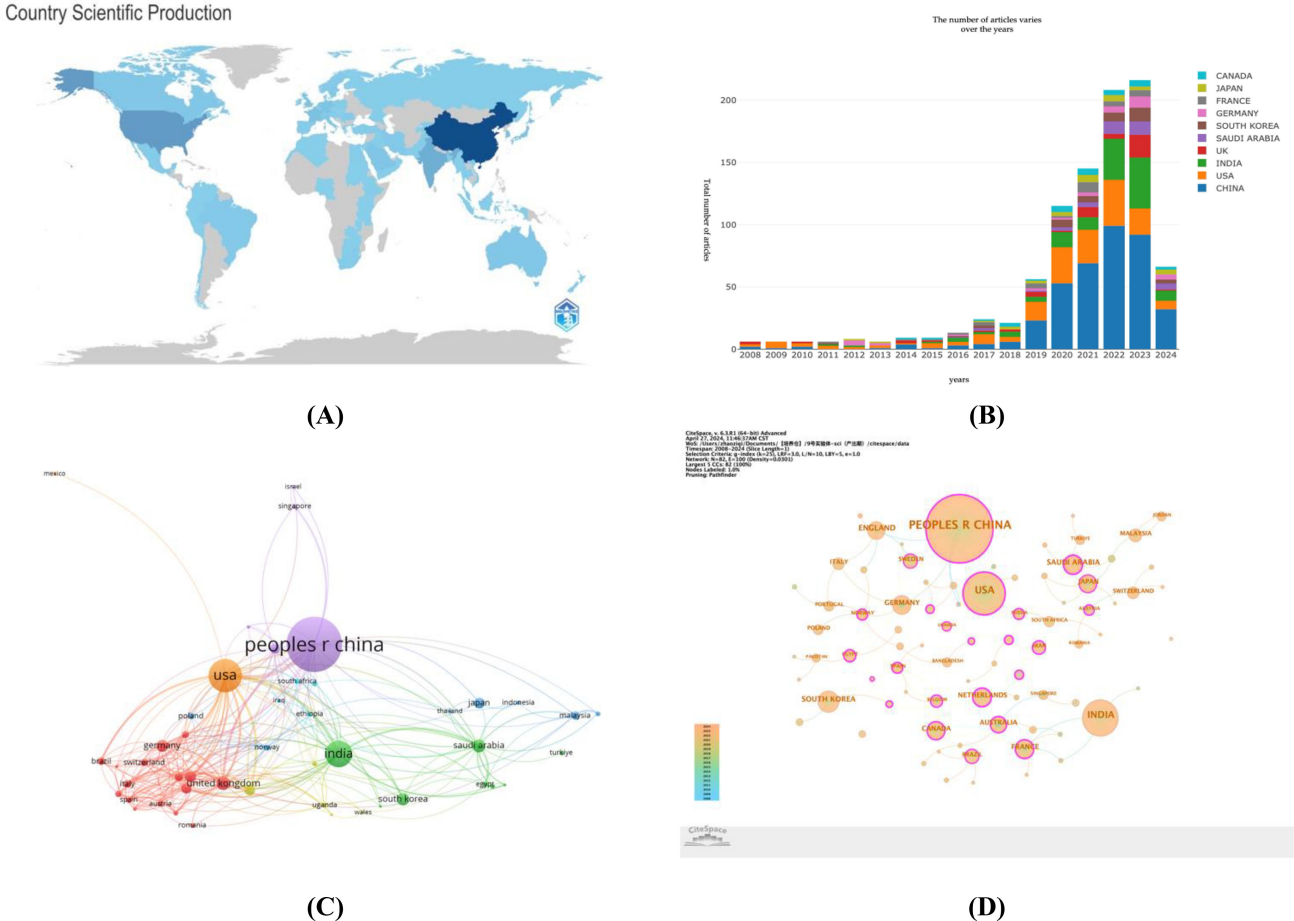
**(A)** World map based on the total publications of different countries/regions; **(B)** The changing trend of the annual publication quantity in the top 10 countries/regions over the past 20 years; **(C)** Citation network visualization map of countries/regions; **(D)** Map of the centrality of national cooperation.

Researchers spanning 81 countries worldwide have collectively contributed valuable academic achievements in the interdisciplinary research of cervical cancer and artificial intelligence. The geographical distribution of these contributions is clearly illustrated in the map presented in **Figure 3A**. Among the countries with more than 100 publications, China, the United States, and India stand out particularly.

**Figure 3B** further reveals the annual publication trends of the top 10 countries, with China leading in publication volume with 392 articles, while the United States tops the influence rankings with a total of 5,351 citations and a Total Link Strength (TLS) of 159.

The application of the VOSviewer tool (as seen in **Figure 3C**) has enabled an in-depth analysis of the research collaboration network between countries. Under the criterion of having published at least four articles, a total of 49 countries were involved. The thickness of the lines in the diagram directly reflects the closeness of the co-authorship relationships between countries, with thicker lines indicating stronger collaborations. Based on the ranking of Total Link Strength (TLS), the United States, China, India, the United Kingdom, and Germany are in the top five.

The use of CiteSpace software has further revealed the centrality of countries within the collaboration network (**Figure 3D**). Nodes marked with a purple circle in the diagram have a centrality value exceeding 0.1, indicating that these countries play a more critical role within the network. Although Iran has a high centrality value of 0.75, indicating its active role in collaborations, it did not appear in the publication volume ranking due to not meeting the threshold for the top 10 countries in terms of total publications. **Table 1** provides additional explanations.

**Table 1:** Ranking of the top 10 countries in volume and centrality of publications.

Sorted by the Number of Publications					Sort by Centrality				
Rank	Country	Count	Centrality	Citations	Total Link Strength	Rank	Country	Centrality	Count
1	China	392	0.30	5172	101	1	Iran	0.75	14
2	USA	173	0.25	5351	159	2	Austria	0.61	10
3	India	120	0.05	1378	72	3	Belgium	0.61	8
4	England	42	0.08	588	63	4	Netherlands	0.53	26
5	Saudi Arabia	37	0.43	697	48	5	Iraq	0.50	4
6	South Korea	36	0.05	451	12	6	Saudi Arabia	0.43	37
7	Germany	32	0.08	650	49	7	Denmark	0.43	4
8	France	29	0.21	1119	43	8	Norway	0.39	11
9	Japan	28	0.14	653	44	9	Canada	0.38	28
10	Canada	28	0.38	423	6	10	Australia	0.37	24

### 3.3 Productive institutions analysis

In our in-depth analysis of the field where cervical cancer research intersects with artificial intelligence, we included 1,718 institutions that participated in the research. The Chinese Academy of Sciences leads among Chinese institutions with 32 publications, followed by Chinese Academy of Medical Sciences (27 publications) and the National Institutes of Health (NIH) of the United States (24 publications). However, publication volume does not always correspond to institution Total Link Strength (TLS). According to the data in **Table 2**, the top three institutions in terms of TLS are the National Cancer Institute of the National Institutes of Health (TLS=141), Southern Medical University (TLS=81), and the Chinese Academy of Medical Sciences (TLS=80).

**Table 2:** Ranking of the top 10 institutions by number of publications.

Rank	Organization	Documents	Centrality	Citations	Average Citation	Total Link Strength	Country
1	Chinese Academy of Sciences	32	0.08	151	4.72	44	China
2	Chinese Academy of Medical Sciences	27	0.02	344	12.74	80	China
3	National Institutes of Health (NIH)	24	0	615	25.63	43	USA
4	Peking Union Medical College	23	0.06	344	14.96	80	China
5	University of Texas System	23	0.06	173	7.52	47	USA
6	Huazhong University of Science & Technology	20	0.02	296	14.80	32	China
7	NIH National Cancer Institute (NCI)	19	0.15	834	43.89	141	USA
8	Southern Medical University	19	0	316	16.63	81	China
9	China Medical University	18	0	593	32.94	61	China
10	Wuhan University	14	0.03	181	12.93	43	China

**Figure 4:**
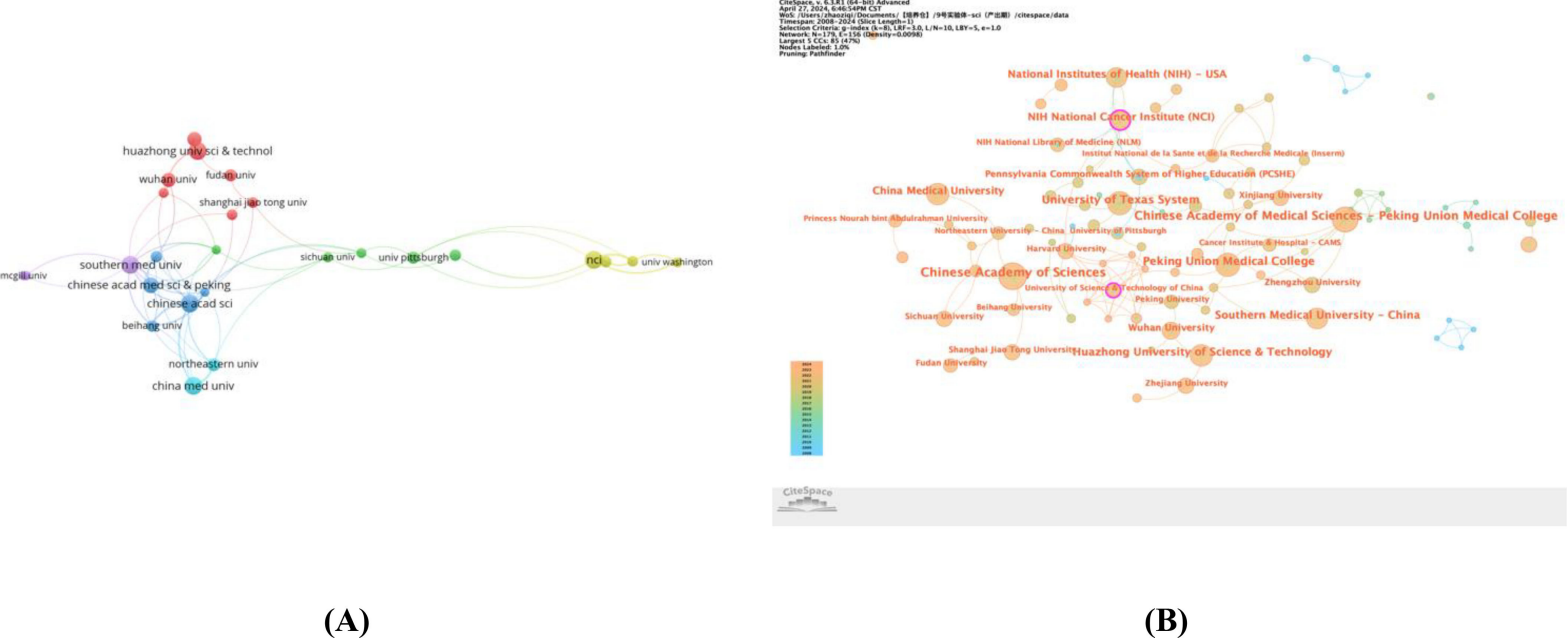
**(A)** The visualization map of institutions co-authorship analysis generated by VOSviewer; **(B)** Centrality map of institutional collaboration.

Despite collaborative efforts, most collaborations between institutions are relatively scattered, with institutions in the United States and China frequently collaborating within their own countries (as shown in **Figure 4A**). To delve into the inter-institutional collaboration network, we utilized CiteSpace software, setting a time span from 2008 to 2024, with a 1-year slicing length, using institutions as the node type, and setting the g-index to k=8, to generate an institutional collaboration graph with 179 nodes and 156 edges (Density=0.0098). **Figure 4B** particularly highlights institutions with significant influence in the field of cervical cancer and artificial intelligence research. In the graph, the outermost purple circle around nodes indicates a centrality value exceeding 0.1, representing a notable contribution to the field by these institutions. Both the National Cancer Institute (NCI) of the National Institutes of Health and the University of Science and Technology of China are encircled in purple, with centrality values of 0.15 and 0.12, respectively, demonstrating their leading positions in this domain.

### 3.4 Authors and co-authors analysis

**Figure 5:**
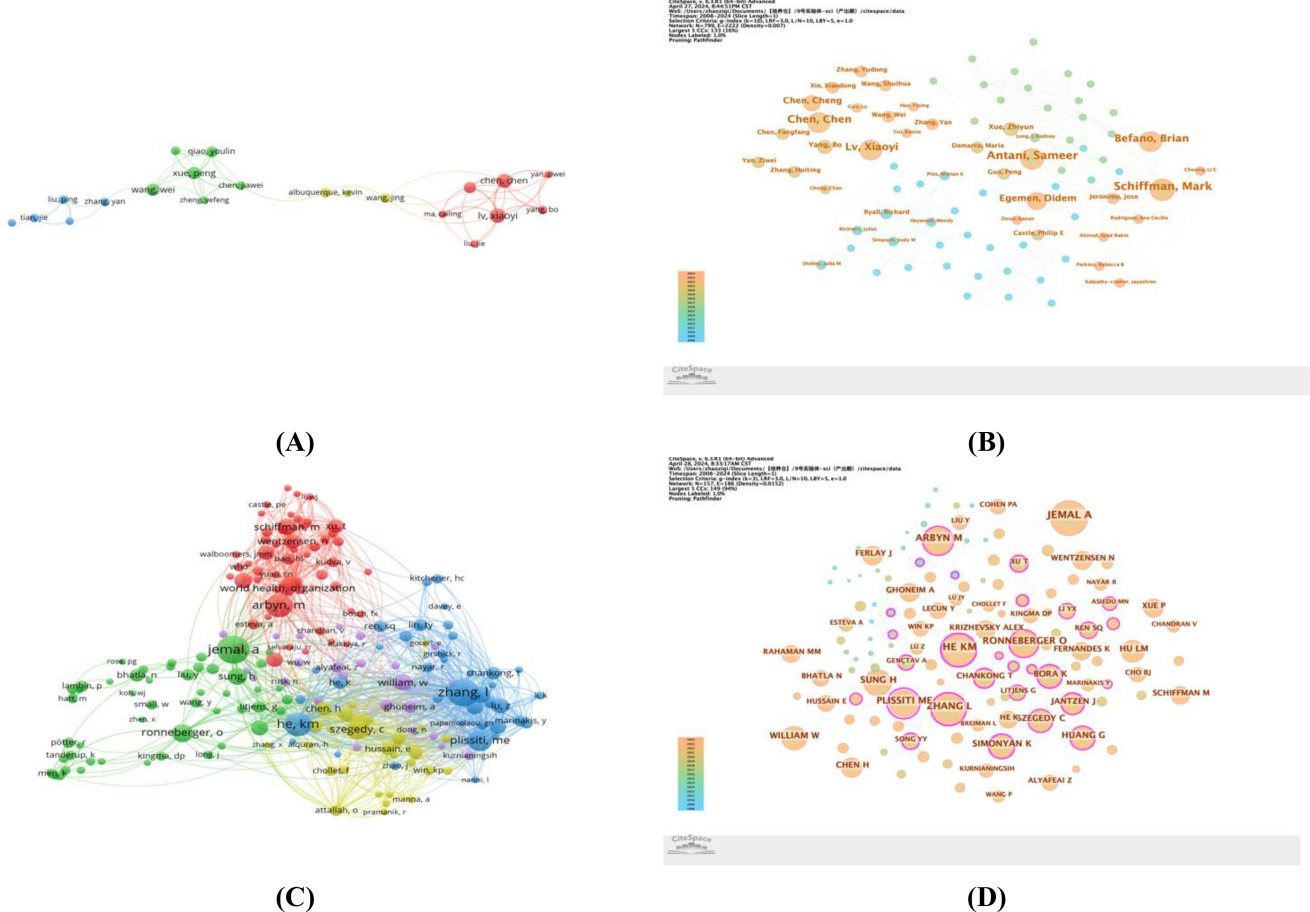
**(A)** Visualization map of co-authorship; **(B)** Centrality map of author collaboration; **(C)** Visualization map of co-citation analyses of authors; **(D)** Map of the centrality of co-citation authors.

In the broad examination of this study, we identified 5,229 authors who have directly contributed articles, as well as 20,868 scholars who have been widely co-cited in the literature. **Table 3** specifically highlights the top ten authors by publication volume and the top ten co-cited authors by citation frequency. Schiffman, Mark leads with 15 publications, followed by Antani, Sameer with 13 publications, and Lv, Xiaoyi with 12 publications. Nevertheless, **Figures 5A, B** show that the centrality values of all authors do not exceed 0.1, and thus no purple circles appear in the collaboration network graph, suggesting that the current visible collaborations are not yet extensive.

**Table 3:** The 10 most productive authors and the top 10 co-cited authors with the highest citations.

Rank	Author	Country	Count	Citations	Total Link Strength	Co-cited Author	Citation	Centrality	Country
1	Schiffman, Mark	USA	15	434	68	Jemal, A	181	0.06	USA
2	Antani, Sameer	USA	13	429	57	Zhang, L	175	0.01	China
3	Lv, Xiaoyi	China	12	144	25	He, Km	143	0.13	China
4	Befano, Brian	USA	11	304	61	Arbyn, M	136	0.08	Belgium
5	Chen, Chen	China	11	120	24	Plissiti, Me	119	0.16	Greece
6	Li, Chen	China	9	344	19	Szegedy, C	103	0.01	France
7	Wang, Wei	China	9	84	5	Ronneberger, O	93	0.10	Germany
8	Xue, Peng	China	9	213	15	Sung, H	87	0.04	USA
9	Xue,Zhiyun	China	9	392	37	Simonyan, K	84	0.02	USA
10	Chen, Cheng	China	8	55	19	Song, Yy	82	0.02	Singapore

Further analysis of the co-citation author network reveals (**Figures 5C, D**) that Jemal, A, Zhang, L, and He, Km occupy the top three positions in citation frequency with 181, 175, and 143 citations, respectively. Within this network, Plissiti, Me, He, Km, and Ronneberger, O have centrality values reaching or exceeding 0.1, specifically 0.16, 0.13, and 0.1, indicating that they play key roles in the research field of cervical cancer and artificial intelligence. The co-citation author collaboration network diagram clearly depicts a close and mature collaborative system, reflecting the strong cooperative relationships that have been established among scholars in this field.

### 3.5 Top journals analysis

**Figure 6:**
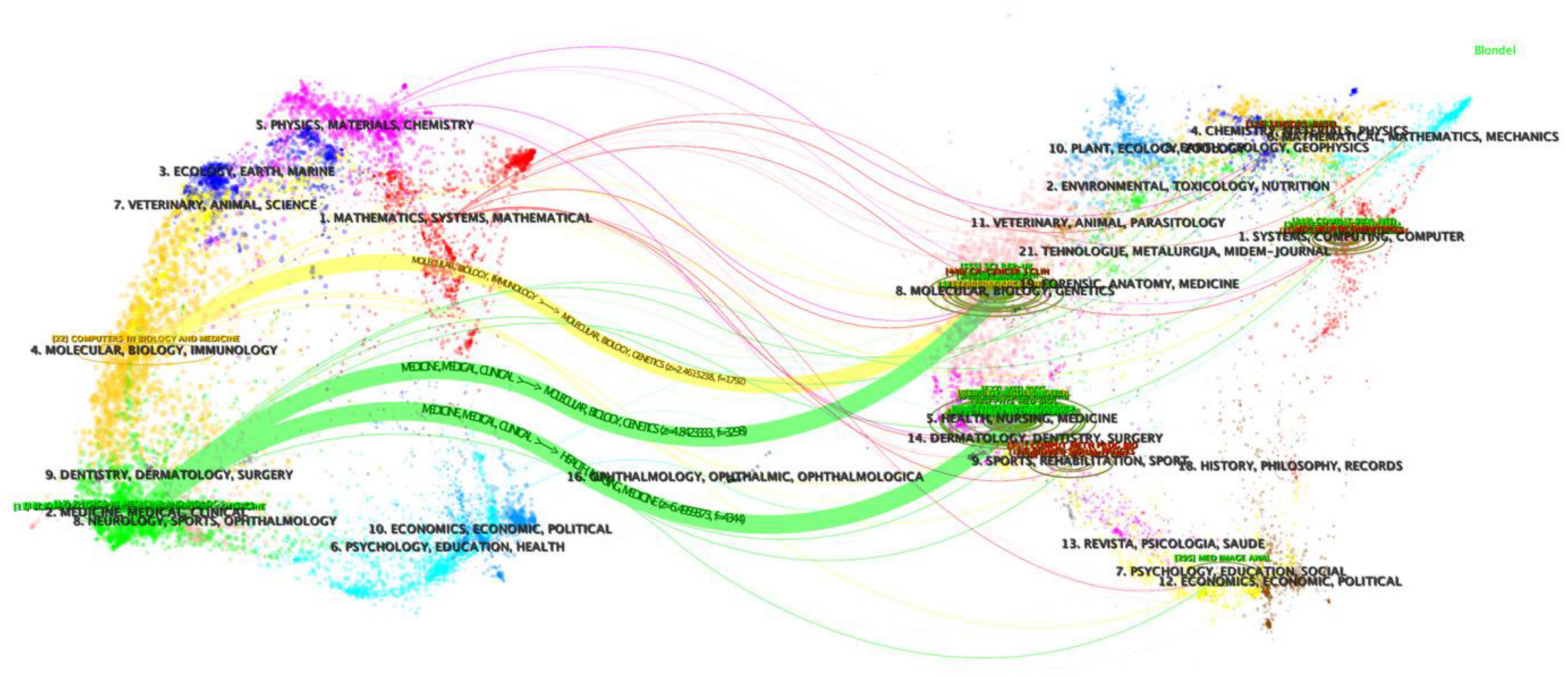
A dual-map overlap of journals with studies researching artificial intelligence in cervical cancer.

The publications of this study are spread across 373 academic journals. **Table 4** provides a detailed list of the top 10 journals by publication volume, covering key indicators such as the number of papers, country affiliation, impact factor, Journal Citation Reports (JCR) ranking, total citation count, and Total Link Strength (TLS). Among them, “Diagnostics” leads with 34 publications, closely followed by “Frontiers in Oncology” and “IEEE Access” with 32 publications each. Within these journals, two are dedicated to oncology (“Frontiers in Oncology” and “Cancers”), and three focus on nuclear medicine (“Physics in Medicine and Biology,” “Journal of Applied Clinical Medical Physics,” and “Medical Physics”), with most journals positioned in the Q2 category of the JCR, reflecting their academic impact. **Figure 6**, through a dual-layered map, displays the citation relationships between journals, revealing three main citation pathways. The cited literature is predominantly concentrated in disciplines such as molecular biology, immunology, and clinical medicine, while the citing literature is often found in fields including molecular biology, genetics, health care, and medicine. This indicates the breadth and depth of interdisciplinary research at the intersection of cervical cancer and artificial intelligence.

**Table 4:** Top 10 journals related to research on artificial intelligence in cervical cancer.

Rank	Journal Title	Articles	Country	IF	JCR	Total Citations	Total Link Strength
1	Diagnostics	34	Poland	3.6	Q2	186	359
2	Frontiers in Oncology	32	Switzerland	4.7	Q2	351	154
3	IEEE Access	32	USA	3.9	Q2	485	356
4	Biomedical Signal Processing and Control	23	England	5.1	Q2	248	197
5	Computers in Biology and Medicine	22	USA	7.7	Q1	327	183
6	Scientific Reports	22	England	4.6	Q2	422	188
7	Cancers	17	Switzerland	5.2	Q1	89	84
8	Physics in Medicine and Biology	17	England	3.5	Q2	478	81
9	Journal of Applied Clinical Medical Physics	13	USA	2.1	Q3	211	57
10	Medical Physics	12	USA	3.8	Q2	124	54

### 3.6 Top cited references and co-citation references analysis

This analysis encompasses 927 relevant academic publications, of which 139 have been cited more than 30 times. According to the data in **Table 5**, the most cited paper is the one published by Berner, ES, et al. in 2008, which discusses the issue of overconfidence in medical diagnostic errors in the ‘American Journal of Medicine’. It places a particular emphasis on the application of artificial intelligence in cervical cancer screening and how the use of automated assistive screening systems can enhance the accuracy of interpreting cervical cytology smears, with a cumulative citation count of 628. The second most cited is the study by Hatt, M, et al., which delves into the application of PET/CT image texture analysis in quantifying tumor uptake heterogeneity, and analyzes its potential, issues, and future directions in clinical research, garnering 313 citations.

**Table 5:** Top ten cited articles on artificial intelligence in cervical cancer.

Rank	Author	Journal	DOI	Year	Citations
1	Berner, ES; et al.	American Journal of Medicine	10.1016/j.amjmed.2008.01.001	2008	628
2	Hatt, M; et al.	European Journal of Nuclear Medicine and Molecular Imaging	10.1007/s00259-016-3427-0	2017	313
3	Scarinci, IC; et al.	Cancer	10.1002/cncr.25065	2010	217
4	He, L; et al.	Computer Methods and Programs in Biomedicine	10.1016/j.cmpb.2011.12.007	2012	216
5	Hu, LM; et al.	JNCI-Journal of the National Cancer Institute	10.1093/jnci/djy225	2018	210
6	Maspero, M; et al.	Physics in Medicine and Biology	10.1088/1361-6560/aada6d	2018	169
7	Chankong, T; et al.	Computer Methods and Programs in Biomedicine	10.1016/j.cmpb.2013.12.012	2014	168
8	Dunn, AG; et al.	Journal of Medical Internet Research	10.2196/jmir.4343	2015	159
9	Dunn, AG; et al.	Vaccine	10.1016/j.vaccine.2017.04.060	2017	153
10	Ijaz, MF; et al.	Sensors	10.3390/s20102809	2020	150

**Figure 7:**
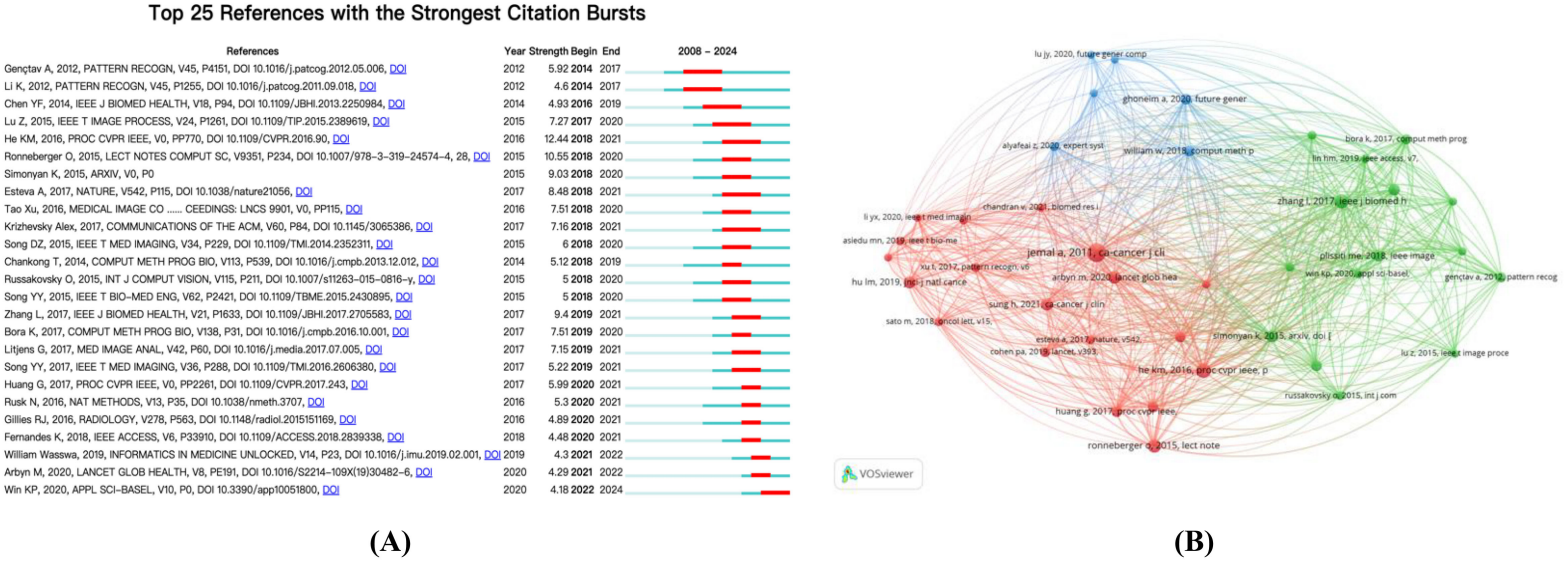
**(A)** Visualization map of top 25 references with the strongest citation bursts from 2008 to 2024; **(B)** The visualization map of references co-citation analysis generated by VOSviewer.

**Figure 7A** further reveals the citation growth trend of the top 25 node documents in this field. The robust citation growth of these documents reflects current research hotspots. Since 2014, citation activity in this field has begun to rise significantly, peaking between 2018 and 2020. This trend indicates that the study combining cervical cancer with artificial intelligence has garnered widespread attention and discussion within the academic community.

In total, the articles cited 29,462 references, with 40 documents frequently cited at least 30 times. Using VOSviewer software, we conducted a co-citation analysis and visualized it (as detailed in **Figure 7B**), discovering that three closely related clusters had formed. These clusters focus on applying cutting-edge image processing and machine learning technologies to enhance the detection and classification capabilities of cervical cancer, while emphasizing the importance of automated analysis of cervical cell images for improving the accuracy and efficiency of screening (21).

The red cluster specifically discusses the VGG19 (22, 23) and CaffeNet (24) models, delving into their performance metrics and focusing on comparing their classification accuracy with existing technologies. The green cluster proposes an innovative ensemble approach, utilizing a voting strategy (25), highlighting its advantages in scalability and practicality. The blue cluster, on the other hand, focuses on the DeepCervix (26) framework and hybrid deep feature fusion techniques, discussing the application of the SIPaKMeD and Herlev datasets, and reporting in detail on the results of classification accuracy (3, 27).

### 3.7 Keywords analysis

**Figure 8:**
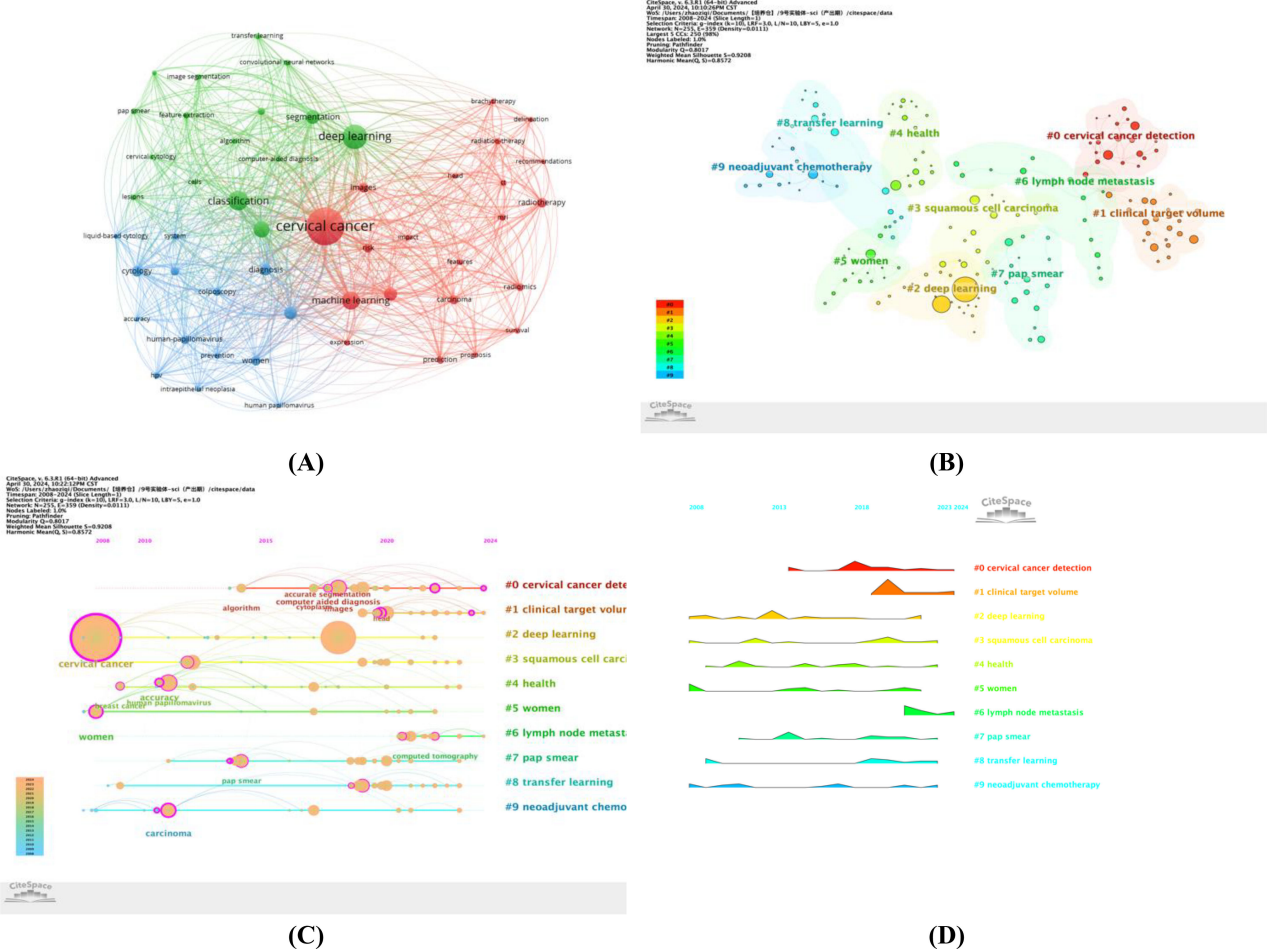
**(A)** Visualization map of keywords generated by VOSviewer; **(B)** Keywords Cluster analysis map; **(C)** CiteSpace visualization map of timeline view; **(D)** CiteSpace visualization map of landscape view.

Keyword analysis has revealed the interconnections between research topics and reflects the hotspots and trends within specific research areas. In this study, we conducted an in-depth analysis of 3,506 keywords and found that 51 keywords were cited more than 20 times. Using VOSviewer software, we constructed a map that displays the co-occurrence of keywords (as shown in **Figure 8A**). The size of the nodes in the map is proportional to the frequency of keyword occurrence, while the thickness of the lines between nodes represents the strength of their association; the thicker the line, the higher the co-occurrence frequency of the two keywords and the closer their relationship. Among the numerous keywords, ‘cervical cancer’ has the largest node, followed by ‘deep learning,’ ‘classification,’ and ‘machine learning’.

Furthermore, CiteSpace software was employed to generate a keyword clustering map (see **Figure 8B**). The modularity value Q and the average silhouette value S are important indicators for assessing the significance of clustering. The Q value of this study is 0.8017, which is significantly higher than the benchmark of 0.4, indicating significant clustering structure; the S value is 0.9208, exceeding 0.5, which means the clustering is efficient and has network homogeneity, with tight connections between keywords, making the results persuasive. **Figure 8B** displays 10 clusters, and **Table 6** further describes the specific characteristics of each cluster, with ‘cervical cancer detection’ and ‘clinical target volume’ being the two largest clusters.

**Table 6:** Details of the top 10 keyword clusters.

Cluster	Size	Silhouette	Year	Top Terms (LLR)
#0 cervical cancer detection	23	0.915	2018	cervical cancer detection, convolutional neural network, cervical cancer, medical image processing, ensemble learning
#1 clinical target volume	23	0.948	2021	clinical target volume, radiotherapy, automatic segmentation, cervical cancer radiotherapy, auto-segmentation
#2 deep learning	22	0.923	2013	deep learning, cervical cancer, cervical cancer screening, overall survival, tumor segmentation
#3 squamous cell carcinoma	18	0.828	2016	squamous cell carcinoma, radiomics, immunotherapy, miRNA, metastasis
#4 health	18	0.945	2015	health, deep learning, prevention, screening, human papillomavirus
#5 women	17	0.906	2014	women, deep learning, hpv DNA, high risk, disease
#6 lymph node metastasis	16	0.951	2021	lymph node metastasis, magnetic resonance imaging, ovarian cancer, meta-analysis, tumor delineation
#7 pap smear	16	0.892	2017	pap smear, oversampling, image analysis, neural networks, computer-aided diagnosis
#8 transfer learning	16	0.976	2017	transfer learning, convolutional neural networks, image segmentation, feature extraction, pathology
#9 neoadjuvant chemotherapy	16	0.979	2014	neoadjuvant chemotherapy, single nucleotide polymorphisms, predictive model, locally advanced cervical cancer, random forest

To deeply analyze the keywords and their development trends in this research field, we created a keyword timeline map (**Figure 8C**) and a keyword time peak map (**Figure 8D**). By observing the changes of each cluster over time, a deeper understanding of the key research themes within the field can be achieved. **Figure 8C** shows 10 clusters, numbered from 0 to 9, with each number representing the start and end times of a cluster. The size of the nodes reflects the frequency of keyword occurrence within the clusters, and the colored lines show the co-occurrence relationships between different clusters. It can be seen that clusters #2, #3, #5, and #9 emerged earlier and have continued to receive attention. Current research hotspots include ‘lymph node metastasis,’ ‘cervical cancer detection,’ and ‘clinical target volume.’ **Figure 8D** shows that ‘clinical target volume’ peaked in 2019 and, although it has declined since then, it remains a focus of attention. The clusters that continue to be popular include ‘cervical cancer detection,’ ‘clinical target volume,’ and ‘lymph node metastasis’.

## 4 Discussion

Over the past two decades, artificial intelligence has advanced rapidly and quickly expanded into the field of clinical medicine, particularly in disciplines that are highly dependent on imaging. Cervical cancer research has clearly benefited from the progress of AI technology. Unlike traditional literature review methods, bibliometrics focuses on systematically reviewing the literature of a research field through quantitative means combined with visualization tools. This approach can present complex data in an intuitive way, thereby mapping out the patterns of progress in the research field and predicting future research directions.

This study represents the first attempt to summarize the application of AI in cervical cancer research using bibliometric methods. We employed three tools from bibliometrics: CiteSpace, VOSviewer, and the Bibliometrix R package, using their powerful visualization capabilities to deeply showcase the current research hotspots and potential future development trends.

By reviewing the developmental history of the interdisciplinary field where cervical cancer meets artificial intelligence and examining the annual publication volume and total citation counts, we can observe the evolutionary trends in both the quantity and quality of AI’s role in cervical cancer research. In the early stages of research, the annual publication volume was approximately maintained at around 10 articles, until after 2018 when the field experienced explosive growth, a phenomenon that reflects the significant contributions and knowledge accumulation from early studies to the subsequent rapid development.

China leads globally in research contributions to this field, with its continuous growth in publication volume demonstrating the country’s emphasis on scientific research (as shown in **Figure 3A**). Despite leading in the number of publications, China’s total citation count is 5,172 times, slightly lower than that of the United States with 5,351 times. Furthermore, China’s centrality value is 0.3, which is lower than Saudi Arabia’s 0.43. This suggests that although China excels in the quantity of publications, it still needs to strengthen its output of high-quality research and international collaboration. This may be related to China’s relatively late start in the field of artificial intelligence research. According to **Table 1**, Iran ranks first with a centrality value of 0.75, even though its publication volume is only 14 articles. This data indicates the close cooperation of Iran with the global research community and the significant impact of its research findings on a global scale.

The work of research institutions is often deeply influenced by the country to which they belong. According to the data in **Table 2**, out of the top 10 institutions globally by publication volume, seven are from China. This reflects the increasing prominence of China in the field of research that combines cervical cancer with artificial intelligence and its gradual emergence as a significant center for this research theme.

Total Link Strength (TLS) is an important metric for assessing the intensity of collaboration between institutions. Despite the prominent performance of Chinese institutions in terms of publication volume, the National Cancer Institute (NCI) of the United States leads in TLS. This suggests that China needs to continue to strengthen its collaboration with other leading international institutions, by importing and absorbing advanced international technologies and theories, to enhance its global impact in the scientific research field.

At the same time, inter-institutional collaboration is generally constrained by geographical location. As shown in **Figure 4A**, institutions in the United States and China interact more within their own countries, implying that there is further work to be done in promoting international cooperation and coordination.

Through the analysis of the author collaboration network, we found that none of the authors had a centrality value exceeding 0.1 (as shown in **Figure 5B**), indicating that collaboration among authors is not frequent, which is also reflected in the distribution of nodes in **Figure 5A**. Although the majority of the top 10 authors by publication volume are from China, authors from the United States hold a place in the top two spots, demonstrating their significant academic influence. Particularly noteworthy is Schiffman, Mark, who has the highest publication volume and whose research work is primarily focused on the field of cervical cancer screening. He is committed to exploring innovative screening technologies and methods. In these studies, Schiffman, Mark has extensively applied artificial intelligence technologies, especially deep learning algorithms, to enhance the accuracy and efficiency of screening. Through various projects, his team has developed and tested automated image analysis tools capable of processing image data in cervical cancer screening (28–30), assisting in the identification and classification of cervical cancer or its precancerous lesions (29, 31).

Regarding co-cited authors (as shown in **Table 3**), Jemal, A ranks first with the highest citation frequency, but his centrality value does not reach 0.1 and is not marked with a purple circle in **Figure 5D**. Jemal’s research covers global cancer statistics, including various types of cancer such as lung, breast, and colorectal cancer, and analyzes cancer incidence and mortality rates across different regions of the world (32). Additionally, the research explores cancer prevention and control strategies, with a particular focus on interventions targeting major risk factors, such as smoking, weight issues, and infections (33–35). Among the top 10 authors by co-citation frequency, Plissiti, ME has the highest centrality value, reaching 0.16. Her research is focused on developing and evaluating methods for the automatic detection and segmentation of cell nuclei in Pap smears (36, 37), which is crucial for improving the automation and accuracy of cervical cancer screening.

In the academic publishing field of the intersection between cervical cancer and artificial intelligence research, according to the data in **Table 4**, ‘Diagnostics’ (IF=3.6, Q2), ‘Frontiers in Oncology’ (IF=4.7, Q2), and ‘IEEE Access’ (IF=3.9, Q2) are the three journals with the highest number of published articles. The Impact Factor (IF), Journal Citation Reports (JCR) category, total citation count, and Total Link Strength (TLS) are key indicators for evaluating the academic level of a journal. ‘IEEE Access’, with the highest total citation count among the top 10 journals by publication volume, demonstrates its significant position in this academic field and suggests that these journals may prioritize publishing more papers on the application of artificial intelligence in cervical cancer research in the future.

At the same time, ‘Diagnostics’, ‘Frontiers in Oncology’, and ‘IEEE Access’, as journals with a high volume of publications, are expected to continue publishing high-quality research results and contribute to the development of this field. The citation of papers shows that the cited literature is largely concentrated in disciplines such as molecular biology, immunology, and clinical medicine, while the articles that reference this literature are widely found in fields including molecular biology, genetics, health care, and medicine. This reflects the interdisciplinary breadth and depth of research at the intersection of cervical cancer and artificial intelligence and indicates that this research field continues to receive widespread attention from both the academic and medical communities.

By examining the top 10 highly cited publications related to the application of artificial intelligence in the field of cervical cancer, we can gain insights into the research hotspots and development trends of the field. These papers generally focus on using artificial intelligence technology to improve cervical cancer screening techniques, covering automated image analysis, deep learning algorithms, and predicting disease risk through machine learning models, while also employing various data-driven methods to enhance the accuracy of predictions (38–40). In **Table 5**, the most cited document is from Berner, ES, et al. (41), which emphasizes the application of artificial intelligence in cervical cancer screening and discusses how automated assistive screening systems can enhance the precision of cervical cytology smear examinations. The article by Mathieu Hatt et al. is the second most cited article (42). It explores the complexity of tumors and the use of PET/CT scans, a non-invasive imaging technique, to assess this complexity. By analyzing the texture of tumors, researchers can better understand their characteristics, such as their invasiveness, likelihood of spreading, and response to treatment. This method has proven valuable for various cancers, including those of the esophagus, lung, rectum, breast, head and neck, and brain. However, implementing texture analysis is complex, involving steps like image acquisition, reconstruction, preprocessing, and segmentation. These steps can vary, leading to inconsistent results. To address this, researchers have turned to machine learning to select key features from the extensive data generated by texture analysis. This not only helps pinpoint the most relevant features but also improves the accuracy and reliability of the findings.

Keyword analysis provides a unique perspective for us to discern the development trajectory and trends of artificial intelligence within the field of cervical cancer. To gain a comprehensive understanding of the hotspots and cutting-edge topics within this field, we utilized the VOSviewer software for a visual analysis of high-frequency keywords (**Figure 8A**). The analysis revealed keywords such as ‘cervical cancer,’ ‘deep learning,’ ‘classification,’ and ‘machine learning,’ which represent the current hot topics of research. Currently, the application of artificial intelligence in the field of cervical cancer is primarily focused on areas such as early diagnosis, treatment plan formulation, prognosis evaluation, and pathological image analysis, with machine learning and deep learning being the most widely used technologies.

Further analysis using the CiteSpace software (**Figure 8B**) led us to conduct keyword clustering, where we found that ‘#0 cervical cancer detection’ and ‘#1 clinical target volume’ are the two largest clusters. This indicates that the application of artificial intelligence in cervical cancer detection and image recognition is relatively mature and is an important research topic in the field. Deep learning algorithms are extensively applied in the analysis of cervical cell images, with the aim of enhancing the accuracy and efficiency of cervical cancer screening. For instance, deep learning algorithms are used for the classification of cervical smear images (43) and in AI-assisted liquid-based cytology (LBC) for cervical cancer screening (44). Additionally, deep learning models are also employed for the automation and optimization of radiation therapy planning, including the automatic segmentation of organs at risk (OARs) and clinical target volumes (CTVs) for cervical cancer patients (45).

The timeline view analysis (**Figure 8C**) further demonstrates that research in the field of cervical cancer with artificial intelligence has consistently revolved around clinical applications. As image processing algorithms have matured, the focus of research has gradually shifted towards the formulation of treatment plans, prognosis evaluation, and pathological analysis. Building on early theoretical research and technical exploration, artificial intelligence has been widely applied in various aspects of cervical cancer research, including computed tomography (CT) imaging, pathological image analysis, and the processing and analysis of genomic data (9, 46, 47). These studies have yielded encouraging results and provided accurate guidance and support for clinical early diagnosis and treatment decision-making.

**Figure 9:**
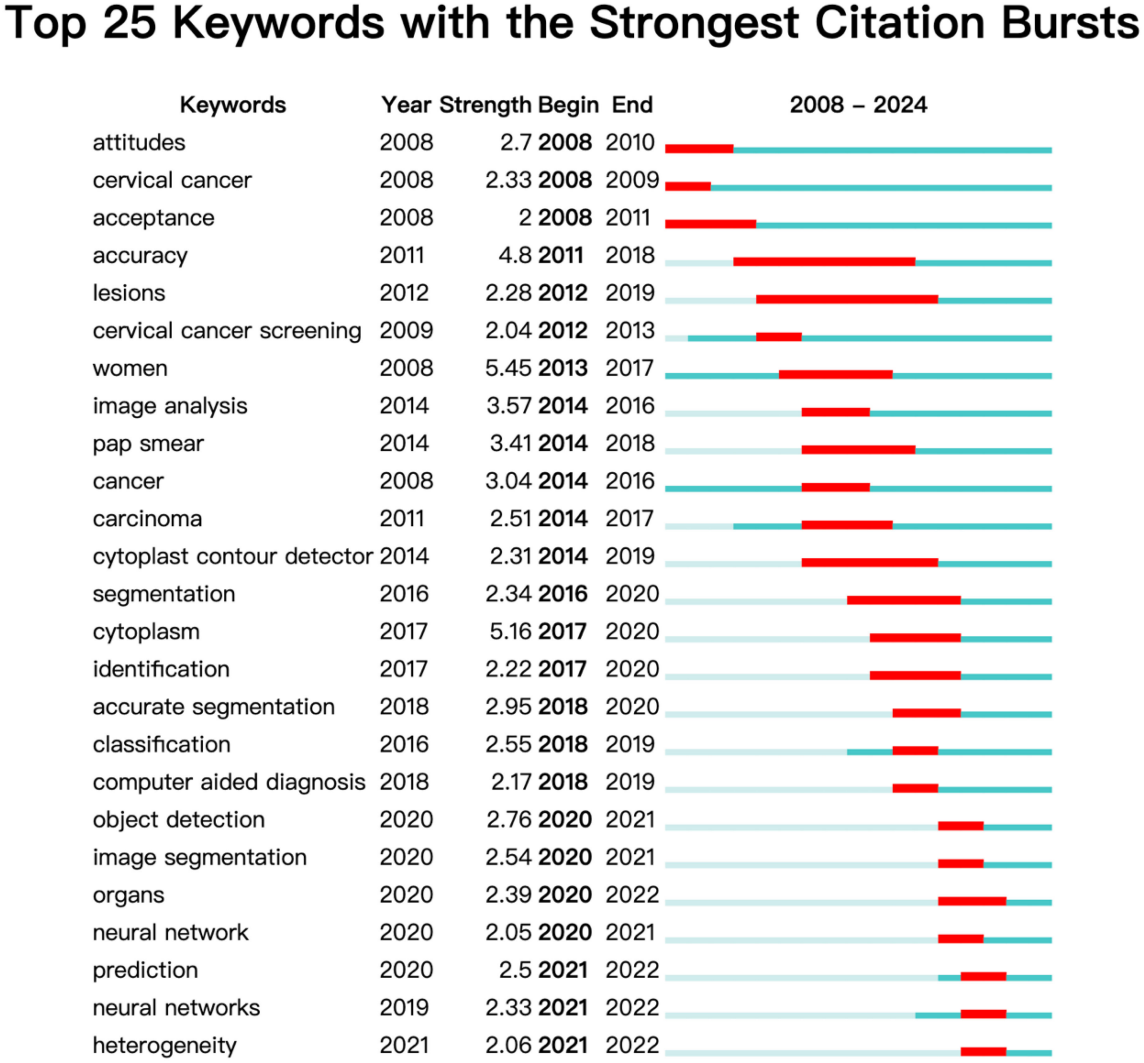
Visualization map of top 25 keywords with the strongest citation bursts from 2008 to 2024.

Keyword emergence analysis (**Figure 9**) shows that between 2011 and 2018, there was a relatively slow evolution of keywords, indicating that ‘accuracy’ and ‘lesions’ were the main research focuses of the period. At the same time, several smaller keywords emerged under these two main clusters, such as ‘cervical cancer screening,’ ‘image analysis,’ ‘pap smear,’ and ‘carcinoma,’ suggesting that research is moving towards increasing the automation, accuracy, and clinical applicability of screening. After 2018, research entered a rapid development phase, with emerging terms including ‘classification,’ ‘computer-aided diagnosis,’ ‘image segmentation,’ ‘neural network,’ and ‘prediction,’ reflecting that recent research has concentrated on improving diagnostic accuracy with artificial intelligence, developing predictive models, optimizing treatment plans, and addressing tumor heterogeneity.

In summary, the application of artificial intelligence (AI) in the field of cervical cancer can be broadly categorized into four main research directions. Firstly, AI plays a vital role in the early diagnosis and screening of cervical cancer. The study by Luo, LM and colleagues highlights the clinical utility of the TruScreen system in screening for high-risk human papillomavirus (HPV) positive patients (48). A retrospective analysis of 318 patients revealed the superior sensitivity of TruScreen compared to traditional thin-layer liquid-based cytology testing (TCT), albeit with slightly lower specificity. Additionally, Fan, WL and colleagues developed a predictive tool based on the Cox regression model and nomograms (49), offering a more intuitive approach for physicians and patients to understand the overall survival rate of cervical cancer patients, thereby aiding in clinical decision-making and treatment planning.

Secondly, AI has demonstrated its potential in planning treatment regimens for cervical cancer. Ji-Young Kim and colleagues introduced an online adaptive radiotherapy (oART) technique combined with cone-beam computed tomography (CBCT), as well as an MRI-guided cervical cancer radiotherapy workflow based on a shuttle system (50). Chung, SY, and colleagues developed a deep learning-based automatic segmentation model for the delineation of organs at risk (OARs) and clinical target volumes (CTVs) in cervical cancer radiotherapy patients (45), significantly enhancing the efficiency and accuracy of the segmentation process.

Thirdly, the application of AI in the prognostic assessment of cervical cancer is equally significant. Shi, R and colleagues constructed a prognostic model for cervical cancer by integrating single-cell RNA sequencing and The Cancer Genome Atlas (TCGA) data, effectively evaluating patient prognoses (19). The deep learning radiomics nomogram (DLRN) established by Zhang, YJ (51) and colleagues, which is based on intratumoral and peritumoral regions within MRI images and clinical features, is used to predict the recurrence risk of early-stage cervical cancer and has demonstrated high accuracy in both internal and external validation cohorts.

Lastly, notable advancements have been made in the application of AI in pathological image analysis. The deep integrated feature fusion (DIFF) method proposed by Fang, M and colleagues, which combines convolutional neural networks (CNNs) and vision transformers, has improved the accuracy of cervical cell image classification (52). Li, Z and colleagues utilized a squeeze-and-excitation attention network (SE_AN) model to predict dose distribution in brachytherapy, providing a new avenue to enhance the efficiency and quality of radiotherapy planning (53).

The integration of artificial intelligence (AI) in the field of cervical cancer marks a transformative era, with its capabilities in early detection, treatment personalization, and prognostic evaluation already proving to be of immense value. The technology’s predictive prowess has reached new heights, streamlining clinical diagnosis and refining the accuracy of patient outcome predictions. Looking ahead, the fusion of AI with multi-omics data stands as a beacon of hope, promising to unlock deeper molecular insights into cervical cancer. This integration is anticipated to catalyze the discovery of novel biomarkers and drive the evolution of personalized therapeutic strategies. As AI continues to merge with cutting-edge research, the horizon of cervical cancer care is set to embrace a new paradigm— one characterized by precision, individualization, and an unwavering commitment to patient-specific outcomes. The future of cervical cancer management, empowered by AI, is on the brink of a significant leap forward, offering patients a more customized and effective approach to care.

Despite the significant progress and immense potential demonstrated by artificial intelligence (AI) in the field of cervical cancer research, it still faces several challenges and limitations in practical application. One crucial issue in the application of AI to cervical cancer is the interpretability of models. AI models, particularly deep learning models, are often considered “black boxes” due to the opacity of their decision-making processes (54). This is especially critical in the medical field, where both doctors and patients urgently need to understand the basis on which the models make their decisions. For instance, when an AI model identifies abnormal cells during cervical cancer screening, it is essential for the physician to be able to clearly explain these findings to gain the patient’s trust and understanding. However, the complex structure of deep learning models, which involves a multitude of parameters and layers, makes their decision-making process difficult to interpret. Although interpretability techniques such as feature importance analysis and heatmap visualization exist, they have their limitations in fully elucidating the model’s decision-making process. Moreover, enhancing model performance often involves trade-offs with interpretability, presenting a challenge that requires delicate balancing.

The trust and acceptance of artificial intelligence (AI) technology by patients are key to its successful application in cervical cancer screening. However, the opacity and complexity of AI systems, coupled with the need for large amounts of sensitive medical data during the training process, may raise concerns and unease among patients (55, 56). To enhance patient acceptance of AI technology, effective communication and education are essential, clearly explaining to patients how AI works and how it can improve the accuracy and efficiency of screening. Additionally, ensuring that data collection, storage, and analysis processes comply with data protection regulations such as the General Data Protection Regulation (GDPR), and obtaining explicit consent from patients, is crucial to allow them to fully understand how their data will be used and the benefits and risks associated with AI systems (57).

Protecting patient privacy and data security is another critical aspect of building trust (58, 59). Furthermore, the decision-making process of AI systems is often a “black box,” making the transparency and interpretability of AI systems vital for establishing trust (60). In 2020, the WHO embarked on an 18-month initiative to formulate guidelines, which led to the publication of the “WHO Guidelines on the Ethics and Governance of Artificial Intelligence for Health” in 2021. The document outlines pivotal ethical principles, scrutinizes a spectrum of ethical dilemmas and challenges, and offers strategic governance advice and recommendations. These central ethical tenets encompass clinical value and safety, fairness and equality, accessibility and uptake, transparency and accountability, as well as adherence to regulations (61).

AI systems may produce unfair decision outcomes due to biases in training data, which requires careful consideration in data collection and algorithm design. Determining liability can be very complex when AI systems make errors in diagnosis or treatment decisions, and the boundaries of responsibility between medical professionals, AI developers, and data scientists need to be clarified. AI technology may be used for unethical purposes, such as analyzing patient data to predict their future health status, which could be used for insurance or employment discrimination. Therefore, the WHO guidelines underscore the significance of equity and equality, stipulating that the application of AI technology should not exacerbate health disparities.

To ensure that the application of AI technology does not exacerbate health inequalities, it is necessary to implement strict data protection measures alongside technology deployment to prevent unauthorized access and data breaches. Patient feedback and opinions should be actively considered and incorporated into the development and implementation of AI technology to ensure that technological advancements truly meet the needs of patients. Moreover, the lack of standardized evaluation processes makes it difficult to compare the results of different studies, which not only limits the clinical application of AI technology but may also hinder its further development in the field of cervical cancer screening (62).

With the development of AI technology, continuous regulation and evaluation are needed to ensure that its application in the medical field complies with ethical standards. This may include regular audits and assessments of AI systems to ensure their behavior complies with ethical and legal requirements. By doing so, we can enhance the trust of healthcare professionals and patients in AI technology, promote the integration and innovation of AI technology in the practice of cervical cancer screening, and ultimately achieve the goal of improving screening efficiency and patient health outcomes.

## 5 Limitation

Our study also has several notable limitations. Firstly, the literature search was primarily based on two index categories, SCI-E and SSCI, within the Web of Science Core Collection. Although we carefully selected indexes most relevant to the research topic, this choice may have excluded other important literature from other databases, potentially introducing a selection bias. Additionally, due to the limitations of bibliometric software algorithms, some degree of data filtering and aggregation was inevitable during the parameter setting process, which could have resulted in certain biases, although we endeavored to minimize this as much as possible. Secondly, this study included only English-language literature, which may have overlooked valuable findings in non-English documents, leading to incomplete data. Lastly, as the accumulation of citation counts takes time, our study may not have fully reflected the impact of recently published documents, which could also have affected the accuracy of the assessment results.

Despite these limitations, our research still strives to comprehensively cover existing publications on the application of artificial intelligence in the field of cervical cancer and provides valuable insights into the research hotspots, trends, and future development directions of the field.

## 6 Conclusion

This study employs bibliometric methods to conduct an in-depth analysis of existing publications on the application of artificial intelligence (AI) in the field of cervical cancer. The analysis reveals that AI technology is playing an increasingly important role in several key aspects of cervical cancer, including early diagnosis screening, treatment plan formulation, prognostic evaluation, and image analysis. Currently, the research focus in this field is progressively shifting towards deep learning techniques, with the aim of achieving precise diagnosis and treatment.

Particularly noteworthy is that China and the United States have made significant progress in this research area and are expected to maintain a leading position in the future. However, to further advance the field, strengthening international communication and collaboration is especially important. Establishing closer cooperative relationships with scientific powerhouses like the United States can facilitate the exchange of knowledge and mutual technological advancement.

Moreover, interdisciplinary collaborative research is also a direction worth paying attention to. By integrating professional knowledge and skills from different fields, new perspectives and innovative ideas can be brought to the study of AI applications in cervical cancer. For instance, experts from fields such as computer science, bioinformatics, and clinical medicine can collaborate to develop more efficient and accurate AI models and tools, thereby enhancing the diagnostic accuracy and therapeutic outcomes for cervical cancer.

## Data Availability

The original contributions presented in the study are included in the article/**Supplementary Material**. Further inquiries can be directed to the corresponding author.

## Supplementary material

Supplementary Table 1 Distribution of Research Fields Included in the Literature.
